# Linking Perceived Environmental CSR to Residents’ Environmental Citizenship Behavior in Rural Tourism: The Mediating Role of Resident–Environment Relationship Quality

**DOI:** 10.3390/ijerph192416566

**Published:** 2022-12-09

**Authors:** Xiaojuan Rao, Hongliang Qiu, Alastair M. Morrison, Wei Wei

**Affiliations:** 1 School of Business Administration, Tourism College of Zhejiang, Hangzhou 311231, China; 2 Zhejiang Academy of Culture & Tourism Development, Hangzhou 311231, China; 3 School of Management and Marketing, Greenwich Business School, University of Greenwich, Old Royal Naval College, Park Row, London SE10 9LS, UK; 4 Department of Hospitality Services, Rosen College of Hospitality Management, University of Central Florida, Orlando, FL 32819, USA

**Keywords:** Stimulus-Organism-Response (SOR) model, environmental corporate social responsibility, environmental citizenship behavior, resident-environment relationship quality, environmental identification, environmental commitment, rural tourism, rural destinations

## Abstract

Residents’ environmental citizenship behavior is essential to the environmental protection and sustainable development of rural destinations. However, previous research with regards to environmental citizenship behavior has focused on an employee perspective, rather than a resident one. Through the theoretical lens of the Stimulus-Organism-Response (SOR) model, our research examined how perceived environmental CSR (ECSR) contributes to residents’ environmental citizenship behavior, with resident-environment relationship quality acting as the organism. Data collected from a Chinese rural destination were analyzed with a structural equation modeling approach. Results indicate that: (1) perceived ECSR directly and positively influences residents’ environmental citizenship behavior; (2) relationship quality variables (i.e., environmental identification and environmental commitment) directly and positively affect residents’ environmental citizenship behavior; (3) environmental identification directly and positively affects environmental commitment; (4) relationship quality variables positively mediate the effect of perceived ECSR on residents’ environmental citizenship behavior. The current research complements existing tourism literature on environmental citizenship behavior with a focus on perceived ECSR and relationship quality from the aspect of residents in rural destinations. The findings also provide some practical implications that potentially facilitate the adoption of environmental citizenship behavior among residents for sustainable destination management.

## 1. Introduction

Due to the unique natural and cultural resources in rural destinations, rural tourism has achieved substantial growth in recent years [1,2]. Particularly, since the outbreak of the COVID-19 pandemic, rural destinations have become a preferred option for tourists thanks to their pristine environment and potential health benefits [3,4,5]. The negative impacts of tourists on destinations, however, has become increasingly noticeable as the tourism sector rapidly expands [6,7]. Promoting environmental protection of tourists has become increasingly popular in rural tourism research. In contrast, the research on residents’ environmental citizenship behavior is inadequate.

As another vital stakeholder group for rural destinations, residents live and perform their daily recreational activities at the destination, which are closely related to the environment [8,9]. Residents’ behavior, if not properly restrained, may have more destructive impacts on the environment and public health than temporary tourists, such as soil contamination, water pollution and garbage accumulation [10]. Thus, it is of great value to guide and foster residents’ environmental citizenship behavior for driving sustainable development of the destination. However, the literature has mainly approached environmental citizenship behavior from an employee perspective [11,12]. Little attention has been paid to the perspective of destination residents. Environmental citizenship behavior depicts a balance between an individual’s immediate interests and long-term benefits of the collective or the environment [13]. It stresses the extra-role behavior of the public reflected in their environmental concerns and their irreplaceable roles in environmental conservation [14,15,16,17]. Given that developing residents’ environmental citizenship behavior might generate positive ecological benefits, it is vital to explore the formation of residents’ environmental citizenship behavior in rural destinations.

With its root in environmental management and corporate social responsibility (CSR) [18], environmental CSR (ECSR) is considered as an essential and distinct component of CSR shaped by organizational management strategies [19]. Of all environmental stimuli cues, ECSR is acknowledged as an important stimulating factor. It is identified as a critical predictor of environmental citizenship behavior in organization contexts [11]. However, prior studies have primarily focused on the directed-behavior of employees, rather than residents [20,21,22,23]. To date, little research has examined the links between residents’ perceived ECSR and their environmental citizenship behavior in the destination context. This constitutes the research question of whether perceived ECSR can predict residents’ environmental citizenship behavior. Considering that the destination’s efforts to fulfill its social responsibility potentially prompt residents to behave in a similar manner, it points to the urgent need to examine and validate the effect of residents’ perceived ECSR on their environmental citizenship behavior. Therefore, as a response to the above question, the present research aimed to uncover the potential role of resident’ perceived ECSR as a stimulus in triggering their environmental citizenship behavior in rural destinations.

In spite of the potential values of residents’ perceived ECSR and environmental citizenship behavior in maintaining and improving the environment, the psychological mechanism of how perceived ECSR is converted into environmental citizenship behavior remains challenging for rural destinations. Extant literature on ECSR has identified multiple factors that might bridge the nexus between ECSR and pro-social behavior, such as green practices, personal ties, and brand image [24,25,26]. However, there are few empirical efforts to take the people-environment relationship into account for explaining this conversion process. Human activities can affect environmental quality, while changes in environmental conditions can impact people’s well-being [27]. That is, a reciprocal relationship exists between people and the environment they live in. Empirical studies in environmental psychology have also validated that relationship quality is a significant antecedent to a variety of intentions and behavior [28]. To better understand residents’ decision-making processes of their sustainable actions, it is of great significance to gain new insights into how relationship attributes contribute to the possible effect of residents’ perceived ECSR on their environmental citizenship behavior. This research argues that relationship quality variables (i.e., environmental identification and environmental commitment) may act as imperative mediators of the association between residents’ perceived ECSR and their environmental citizenship behavior. This logic can be theoretically explained as follows. Relationship quality can be recognized as a vital mediator of the above associations. That is, residents’ perceived ECSR can improve the quality of the resident-environment relationship [29,30], which will in turn activate environmental protection [31].

Given the above discussion, our research used the Stimulus-Organism-Response (SOR) model as the theoretical underpinning for explaining the formation of residents’ environmental citizenship behavior. The objectives of this research were to: (1) investigate the relationship between residents’ perceived ECSR and their environmental citizenship behavior; (2) explore the association between resident-environment relationship quality (i.e., environmental identification and environmental commitment) and residents’ environmental citizenship behavior; (3) reveal the correlation between environmental identification and environmental commitment, and (4) examine the mediating role of resident-environment relationship quality. This study contributes to tourism research with a more holistic and nuanced understanding of the relationship between residents’ perceived ECSR and their environmental citizenship behavior. It further highlights the mediating role of resident-environment relationship quality in developing sustainable behavior amongst residents of rural destinations. From a managerial perspective, the findings may offer rural destination managers some helpful implications for promoting residents’ environmental citizenship behavior by enhancing their perception of ECSR practices and the quality of their relationships with the destination environment.

The remainder of this article is structured as follows. Section 2 offers an overall review of the Stimulus-Organism-Response model and proposes hypotheses based on the elucidation of the links between relevant constructs. The methodology and results are presented in Section 3 and Section 4, respectively. Section 5 includes a discussion on the results and presents the theoretical contributions and practical implications of this research. Finally, Section 6 concludes with potential limitations and opportunities for future work.

## 2. Literature Review and Hypotheses Development

### 2.1. Stimulus-Organism-Response Model

Originated from the environmental psychology [32], the SOR model postulates that the external stimuli (S) contained in people’s environment can invoke their internal states (O), which will subsequently exert influence on their behavior (R). Specifically, the perceived social and physical stimuli facilitate evoke people’s cognitive and emotional processes, which thereby trigger their behavioral responses [33]. The SOR model, along with its parsimonious and robust nature, has been widely applied and validated in effectively predicting individual behavior in multiple contexts, such as consumer behavior, tourists’ environmental behavior and, recently, environmental citizenship behavior [11,34,35]. Therefore, our research employed the SOR model to investigate how perceived ECSR (Stimulus) influences residents’ environmental citizenship behavior (Response) through the mediation of resident-environment relationship quality (Organism) in rural destinations.

#### 2.1.1. Stimuli: Environmental Corporate Social Responsibility

A stimulus is described as the influence that activates individual’s psychological changes or behavior [36]. The environmental stimulus contains all elements in people’s environments that can lead to their responses, among which ECSR is regarded as an important element in the organization contexts [11,37]. As an vital component of CSR for reducing the negative effects on the environment [18], perceived ECSR in this research refers to residents’ perception of corporate social responsibility practices for preserving the destination environment, such as whether the environmental programs implemented mitigate the adverse environmental impacts [38]. In this sense, ECSR is a management strategy that may affect residents’ psychological attributes and trigger their behavior. Hence, perceived ECSR is considered as the environmental stimulus in this research.

#### 2.1.2. Organism: Environmental Relationship Quality

Environmental relationship quality manifests the bi-directional relationship between residents and the environment [27]. Environmental identification and environmental commitment have been identified as two critical constructs to measure environmental relationship quality and explain individual environmental behavior [39,40]. Environmental identification emphasizes residents’ psychological connection to the destination [9], which represents the alignment of residents’ perception of the self-concept and the destination image [31]. Environmental commitment, viewing residents and destination as distinct entities, underlines how residents contribute to the destination environment [41]. Environmental commitment embodies the residents’ perceived obligation toward the environment. Organism is described as people’s psychological states stemming from external environmental cues [42]. Since environmental identification and commitment belong to affective attributes of the relationship quality [28], these two relationship quality variables are thus regarded as the organism in the theoretical framework of this research.

#### 2.1.3. Response: Environmental Citizenship Behavior

Response in the SOR paradigm involves eventual outcomes or behavior [35]. Based on the previously defined concept of tourists’ environmental citizenship behavior, environmental citizenship behavior, as the response in the conceptual model of this research, refers to residents’ voluntary behavior for the purpose of improving the destination environment [15]. This concept is described as a single-dimensional construct in some research [43]. However, to gain a deeper understanding of environmental citizenship behavior in various domains, we divided environmental citizenship behavior into private-sphere environmental citizenship behavior (e.g., reducing energy use at home), and public-sphere environmental citizenship behavior (e.g., keeping the surrounding environment clean) [10]. Residents behaving sustainably is crucial to the ecological integrity of rural destinations [27]. Extensive studies have primarily focused on employees’ environmental citizenship behavior through various theoretical lens, such as self-efficacy theory, the SOR model, and the norm activation model [10,11,15]. However, residents’ environmental citizenship behavior remains under-researched, especially how perceived ECSR as the environmental stimulus affects residents’ environmental citizenship behavior in the rural destination setting.

Therefore, this research applied the SOR model to obtain a more comprehensive understanding of environmental citizenship behavior in rural tourism, in which perceived ECSR (as the stimulus) exerts influence on residents’ environmental citizenship behavior (as the response) through environmental relationship quality (as the organism).

### 2.2. Hypotheses Development

#### 2.2.1. Relationships between Stimulus and Organism

Cognitive appraisal theory proposes that people’s subjective assessments of an experience or event will generate emotional responses [27,44,45]. On this basis, when residents perceive the implementation of ECSR practices and receive corresponding benefits from the environment improvement of the destination, they will show gratitude and be more likely to develop a closer relationship with the destination. This relationship will, in turn, enhance residents’ identification with the destination. Marketing literature has indicated that CSR is positively related to customers’ organizational identification [46], while a positive relationship between CSR and employees’ organizational identification has also been reported in organizational research [47,48]. For example, when examining how hotel employees’ perceptions of CSR influence their identification with the hotel, Park and Levy (2014) found that there was a significant positive relationship between CSR and organizational identification.

A key point of cognitive appraisal theory is that a positive association exists between people’s cognition and their emotional reactions [27]. As the perception of the implementation of socially responsible practices in protecting the destination environment, perceived ECSR falls into the category of a cognitive construct. Environmental commitment is viewed as psychological agreement with the natural world, which can be understood as an affective attribute. Despite little evidence being available for supporting the relationship between perceived ECSR and environmental commitment, studies in the marketing domain have confirmed the positive impact of CSR on environmental commitment [20,49]. For instance, in an effort to uncover the underlying mechanism of how CSR is transmitted to positive environmental outcomes in the corporation context, Afsar et al. (2020) confirmed that CSR directly and positively affects employees’ environmental commitment. Thus, this research infers that residents’ perceived ECSR will influence their commitment to the destination environment. According to the above discussions, we formulated the following hypotheses:

**H1.**  *Perceived ECSR directly and positively affects residents’ environmental identification.*

**H2.**  *Perceived ECSR directly and positively affects residents’ environmental commitment.*

#### 2.2.2. Relationship between Relationship Quality Variables

Despite the close relationship between identification and commitment, empirical findings have indicated distinctive differences between these two notions [41]. Particularly, identification emphasizes the match of the organizational image and one’s self-concept, while commitment implies the association between separate psychological entities [41,50]. In the research of organizational behavior, researchers suggest that organization offers employees the tools to work as professionals and endows them with a common identity; organizational identification thus leads to organizational commitment [51]. Existing literature in organizational research offers solid evidence that supports this causal relationship, i.e., employees’ organizational identification has a positive effect on their organizational commitment [47,50,52]. Scholars in environmental management research acknowledge that environmental commitment is a further development of environmental identification [53]. In other words, the level of employees’ environmental identification determines that of their environmental commitment. However, empirical evidence to support this topic is limited in the tourism domain, especially from the aspect of destination residents [9,27]. Given the gradual conversion process from environmental identification to environmental commitment [9], our research argues that the improvement in environmental identification of destination residents will generate a higher level of environmental commitment. Thus, we proposed that:

**H3.**  *Residents’ environmental identification directly and positively affects their environmental commitment.*

#### 2.2.3. Relationships between Organism and Response

As relationship quality theory proposes, building relationships is centered on identifying and addressing the needs of people [54]. From this point of view, one of the effective strategies for encouraging people’s positive behavior is to improve relationship quality [55,56]. In tourism, a healthy environment provides residents with benefits in diverse ways, such as increasing their satisfaction toward life and improving their physical and mental well-being [57,58]. Therefore, it is in the residents’ best interests to preserve the environment to maintain this relationship. Existing literature in marketing has validated that relationship quality plays an essential role in understanding various types of behavior or behavioral intentions, such as purchase intention and behavior [59,60]. In tourism research, relationship quality has also been regarded as an important antecedent of sustainable environmental behavior [55]. Relationship quality is considered as a high-order construct involving multiple dimensions [61]. Recent research on people-environment relationship has identified environmental identification and environmental commitment as two critical constructs in predicting individual environmental behavior [39,40].

Developed from organizational identification, environmental identification in this research is described as residents’ psychological bond to the environment coming from the matches between residents’ perceptions of their own images and the destination images [27]. It is noted that relationship between two parties affects their behavior and leads to extra-role behavior, such as citizenship behavior [62]. It implies that the closer the relationship between residents’ self-concept and the destination, the easier it is for them to adopt environmental citizenship behavior, either in the public domain or private domain. Prior studies have also indicated that people’s identification is closely related to sustainable environmental behavior of both tourists and residents [9,28,62]. Therefore, we assumed that:

**H4.**  *Residents’ environmental identification directly and positively affects their private-sphere environmental citizenship behavior.*

**H5.**  *Residents’ environmental identification directly and positively affects their public-sphere environmental citizenship behavior.*

According to cognitive appraisal theory, emotional reactions lead to associated behavior. Commitment is a psychological state that can determine how people behave [63]. Studies on organizational behavior indicate that as the level of employees’ environmental commitment rises, there are more possibilities for them to adopt favorable behavior to improve the environment [20,63,64]. Empirical findings have also confirmed that environmental commitment can be translated into citizenship behavior for the environment. For example, Daily et al. (2009) identified commitment as one of the key determinants of citizenship behavior directed toward the environment. Further, Abbas et al. (2022) reported that employees’ environmental commitment mediates the relationship between leadership and citizenship behavior for the environment. Therefore, the present research proposed the following hypotheses:

**H6.**  *Residents’ environmental commitment directly and positively affects their private-sphere environmental citizenship behavior.*

**H7.**  *Residents’ environmental commitment directly and positively affects their public-sphere environmental citizenship behavior.*

#### 2.2.4. Relationships between Stimulus and Response

Since ECSR strategies concern environmental issues and responsibility, they are crucial in the cultivation of positive environmental behavior [24]. There is abundant evidence supporting the significance of ECSR in various settings. For instance, in investigating the influence of employees’ perceived ECSR on their workplace environmental behavior, Duarte et al. (2022) noted that employees’ perceptions of ECSR practices directly influence their environmental behavior in the workplace [65]. In an attempt to reveal why Pakistan manufacturing sector employees behave sustainably, researchers obtained similar findings [24]. Moreover, in the green consumption context, Vu et al. (2021) discovered that there is a significant and positive relationship between ECSR initiatives and green purchase intentions among Vietnamese consumers [66]. However, to the best knowledge of this research team, this study is one of the first empirical efforts to explain how perceived ECSR affect environmental citizenship behavior among residents in rural destinations.

According to social exchange theory, social behavior result from cost-benefit consideration with the aim of maximizing the benefits [67]. In the tourism context, a well-preserved environment is the foundation of tourism sustainability, which benefits destination residents in various ways, such as increase of income and employment opportunities [27]. It implies a reciprocal association between the destination and residents. Following this notion, when residents receive long-term benefits from ECSR practices, they will be more inclined to reciprocate and exchange by adopting environmental citizenship behavior in the destinations. That is, perceived ECSR reinforces the reciprocity between the destination and residents. Thus, we proposed that:

**H8.**  *Perceived ECSR directly and positively affects residents’ private-sphere environmental citizenship behavior.*

**H9.**  *Perceived ECSR directly and positively affects residents’ public-sphere environmental citizenship behavior.*

**Figure 1:**
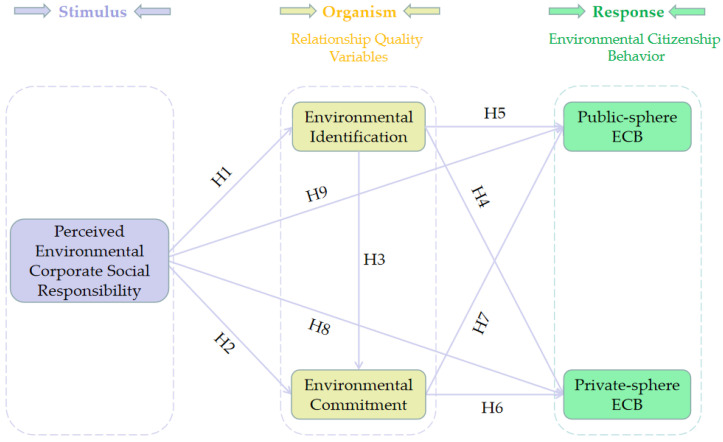
Conceptual framework of the present research.

In light of the above discussions and literature review, we proposed the following conceptual framework for the present research (Figure 1).

## 3. Method

### 3.1. Measurement

Each construct was measured using a variety of items that have been previously verified in the existing literature. Considering the specific research setting, scales of each item were modified. For example, the notion of social responsibility in most CSR literature is approached from a specific organization perspective [19]. Tourism destinations, however, differ from business organizations as they usually involve several forms of businesses, including the provision of food, transportation, travel service and other services [68]. Their activities can influence various stakeholders in the destinations. Therefore, it requires businesses in the destinations to implement ECSR practices to safeguard the environmental integrity of the destinations, which allows the residents to perceive the efforts of these businesses and join the efforts through sustainable acts. Hence, it is noteworthy that perceived ECSR in this research was measured as the perception of a combination of environmentally responsible practices by all component of the destination at large (e.g., ‘This destination implements special programs to minimize its negative impact on the natural environment’). Two sets of three items (e.g., ‘I am very interested in what others think about the natural environment of this destination;’ and ‘In my mind, I am committed to maintaining the greatest environmental interests of this destination’) were adopted from Su et al. (2019) to measure environmental identification and environmental commitment, respectively. Table 1 presents the measurements of all variables. Five-point Likert scales were adopted to evaluate the items, ranging from “strongly disagree” to “strongly agree”.

### 3.2. Pretest of the Measurements

To ensure measurement validity, the scales were translated and back-translated between Chinese and English [69]. Five experts, including two tourism scholars and three destination practitioners, participated in the pre-test for content evaluation. A pilot test was conducted using 55 residents from Yucun Village. The preliminary results reported acceptable reliability (Cronbach’s alpha > 0.70) and validity (standard factor loadings > 0.50) [70,71].

### 3.3. Data Collection and Sample

**Figure 2:**
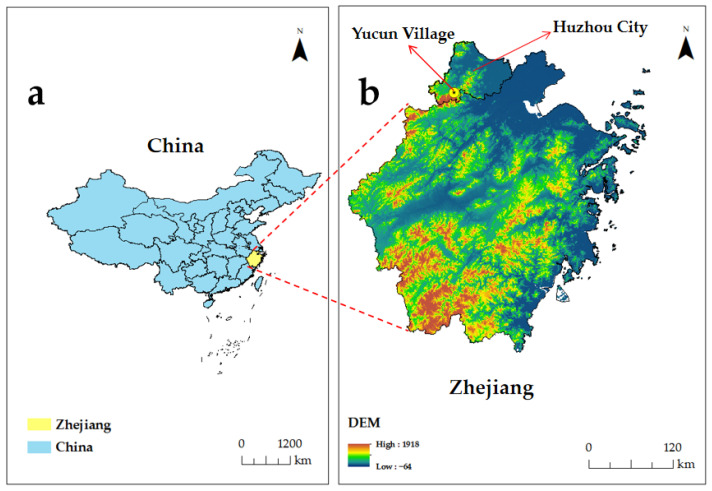
(**a**) Geographical location of Zhejiang Province. (**b**) Geographical location of Yucun Village of Huzhou City.

The data were collected from Yucun Village. This is located in Huzhou City, Zhejiang Province, China. The economy of this village once heavily relied on ore resources, but the price was serious environmental pollution. In recent years, the village started to practice the ecological development concept of “lucid waters and lush mountains are invaluable assets” [72]. The village made great efforts to develop rural tourism by utilizing unique natural resources and landscapes. The change in the development strategy significantly improved environmental quality, residents’ income and local economy. In 2021, the UNWTO recognized it as one of the “Best Tourism Villages” [73]. Statistics show that total tourist arrivals since 2015 have reached over five million [74]. Hence, this research considers it an appropriate site for a field survey as a rural destination. Figure 2 shows the geographical locations of Zhejiang Province and Yucun Village.

The research teams (including three researchers and three trained college students) conducted the field survey in July 2022. Three teams of equal size collected data with the convenience sampling method. After a brief introduction, potential respondents were invited to take part in the survey. Free survey gifts (masks and antiseptic wet wipes) were offered to those who participated. If team members met people who were reluctant to participate or identified themselves as tourists, they would look for other potential respondents. Using this approach, approximately 330 questionnaires were gathered, with 302 valid responses. As suggested by Nunnally (1967), 302 valid responses were adequate since the sample size was over ten times of the total measurement items (17 items in this research) [75]. The valid response rate was 91.5%, with 52.6% of the respondents being males, and 47.4%, females. Regarding the age groups, 19.9% of the participants were below 25, 34.8% between 25–44 years old, 22.8% 45–59 years old, and 22.5% 60 years old and above. Among them, 36.4% received only middle school education or less, 29.5% high school or vocational secondary school education, 34.1% had vocational college education/bachelor degrees and above. In terms of the household annual income, 3% of the households earned less than 50,000 Chinese yuan (not including 50,000 Chinese yuan), 19.2% between 50,000 and 100,000 Chinese yuan (including 50,000, but not including 100,000 Chinese yuan), 23.2%, between 100,000 and 150,000 Chinese yuan (including 100,000, but not including 150,000 Chinese yuan), 20.5% between 150,000 and 200,000 Chinese yuan (including 150,000, but not including 200,000 Chinese yuan), 17.2% between 200,000 and 250,000 Chinese yuan (including 200,000, but not including 250,000 Chinese yuan), 7% between 250,000 and 300,000 Chinese yuan (including 250,000, but not including 300,000 Chinese yuan), and 9.9% more than 300,000 Chinese yuan (including 300,000 Chinese yuan). The univariate skewness values ranged from −0.850 to −0.077, while the kurtosis values ranged from −1.044 to 0.298. All met the skewness and kurtosis requirements [76].

## 4. Data Analysis

### 4.1. Common Method Variance Test

The common method bias (CMB) test is necessary in survey-based research, particularly when the data come from the same source [2,77]. Harman’s single-factor test using the SPSS factor analysis tool showed that no single factor accounted for more than 50 percent of the covariance (the first factor explaining 39.061% of total variance). Confirmatory factor analysis was executed to verify whether a common latent factor explained all the variance. The proposed measurement model showed a much better fit than the common factor model (Δχ^2^(10) = 1261.479, *p* < 0.001). Thus, CMB was not a problem in this research [78,79].

### 4.2. Measurement Model Test

Before the hypotheses test, the confirmatory factor was analyzed to examine the reliability and validity of the constructs, as well as the measurement model fit [80]. The model fit indices (χ^2^/df = 2.843, RMSEA = 0.078, RMR = 0.033, NFI = 0.902, CFI = 0.934, IFI = 0.934, TLI = 0.917, SRMR = 0.0634) suggested that the measurements were good. Table 2 shows that Cronbach’s alpha of each construct varied from 0.776 to 0.909, representing acceptable reliability. We also tested convergent and discriminant validity. The composite reliability values were between 0.786 to 0.911 (Table 2). The standard factor loadings, average variance extracted (AVE), and composite reliability of each construct demonstrated high convergent validity [81]. Discriminant validity was examined by comparing the square root of each construct’s AVE with the correlations between constructs [82]. The statistics in Table 3 provided supporting evidence of discriminant validity. These results showed both the reliability and validity of the measurement mode, which justified further hypothesis testing of the structural model.

### 4.3. Structural Model Test

**Figure 3:**
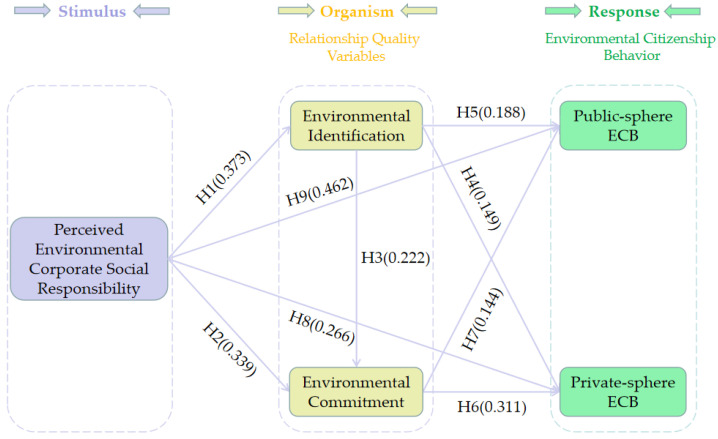
AMOS output results.

The structural equation modeling (SEM) method was employed to test the direct hypotheses. The fit indices (χ^2^/df = 2.824, RMSEA = 0.078, RMR = 0.034, NFI = 0.902, CFI = 0.934, IFI = 0.934, TLI = 0.918, SRMR = 0.0643) indicated a good fit for the structural model. Table 4 shows that all nine hypothesized direct relationships were supported. For example, perceived ECSR directly and significantly affected environmental identification (β = 0.373, *p* < 0.05). Therefore, H1 was supported. Similarly, other eight hypothesized direct relationships (H2 to H9) were also confirmed. The AMOS output results are presented in Figure 3.

### 4.4. Mediating Effect Analysis

We applied the bootstrapping method to examine the mediating effect. Five-thousand bootstrapped samples were generated with a 95% of bias-corrected bootstrap confidence intervals [83,84]. For example, the results in Table 5 suggest a significant specific mediating effect for perceived ECSR on PRECB via EI (95% CI _bias-corrected_: (0.009, 0.123)); 95% CI _percentile_: (0.005, 0.117)). Likewise, the following specific indirect paths were supported: ECSR→EI→EC→PRECB (95% CI _bias-corrected_: (0.008, 0.063)), 95% CI _percentile_: (0.006, 0.057)); ECSR→EC→PRECB (95% CI _bias-corrected_: (0.056, 0.179), 95% CI _percentile_: (0.051, 0.171); ECSR→EI→PUECB (95% CI _bias-corrected_: (0.020, 0.137)), 95% CI _percentile_: (0.018, 0.135); ECSR→EI→EC→PUECB (95% CI _bias-corrected_: (0.002, 0.035)), 95% CI _percentile_: (0.001, 0.030)); and ECSR→EC→PUECB (95% CI _bias-corrected_: (0.008, 0.112), 95% CI _percentile_: (0.004, 0.104).

### 4.5. Explanatory Power of the Conceptual Model

The explanatory power of the model was analyzed by the *R*^2^ of its major endogenous variables [85]. *R*^2^ values of 0.25, 0.09, and 0.01 were the threshold values of large, medium, and small effects, respectively [86]. The findings from the squared multiple correlations indicated that the structural model explained 13.9, 22.0, 32.1, and 40.8% of the variance for EI, EC, PRECB, and PUECB, respectively. These results suggest that the model had sufficient explanatory power with large effects [87].

## 5. Conclusions and Implications

### 5.1. Conclusions

Since environmental citizenship behavior is of great significance to the sustainability of rural destinations, we developed and tested a conceptual model to explore the determinants of residents’ environmental citizenship behavior in rural destinations based on the SOR model. We adopted the SEM method to analyze the data collected from a representative Chinese rural destination. Results indicate that the proposed hypotheses were all supported.

First, the results empirically confirmed the positive effect of perceived ECSR on environmental relationship quality (H1, H2). To be specific, on one hand, there was a positive relationship between perceived ECSR and environmental identification, which is in line with prior empirical findings, i.e., CSR can positively affect customer or employee identification [46,47,48]. On the other hand, perceived ECSR had a positive impact on environmental commitment. This echoes with prior studies that indicated CSR can exert positive influence on employee environmental commitment [20]. That is, when residents perceive that ECSR strategies in the destination bring substantial improvement of the natural environment and benefit them in various ways, they develop a closer bond with the destination and identify themselves with this place, and residents show more dedication to maintaining the ecological integrity of the destination.

Second, this research employed environmental identification and environmental commitment to measure resident-environment relationship quality [9,27]. The relationship between these two constructs was discussed and tested in this research. There are distinctive differences between these two subdimensions of environmental relationship quality, as previous research indicated [41]. Our findings demonstrated that environmental identification is directly and positively associated with environmental commitment (H3). This supports prior research in which employees’ organizational identification was found to determine their commitment to the organization [47,50,52]. Further, it offers new evidence to this positive association by extending it to the rural destination context. This finding implies that a higher match between residents’ self-images and the destination image contributes to a higher level of residents’ commitment to protect the destination environment.

Third, direct and positive relationships between resident-environment relationship quality and residents’ environmental citizenship behavior were confirmed by the results of this research. In support of previous literature [9,62], the results show that residents’ environmental identification is directly and positively related to their environmental citizenship behavior in the private and public domains (H4, H5). Consistent with prior research on employees’ environmental citizenship behavior [20,64], the findings of this study indicate that environmental commitment triggers residents’ environmental citizenship behavior in the private and public domains (H6, H7). These findings underline the importance of environmental identification and environment commitment in driving residents’ private and public environmental citizenship behavior in rural destinations.

Finally, as a vindication of prior research findings in workplace environmental citizenship behavior and consumer green purchase intentions [24,66], results of this research demonstrated that perceived ECSR significantly enhances residents’ private and public environmental citizenship behavior (H8, H9). This means that when residents receive environmental stimuli from the destination (e.g., perceived ECSR), they respond to this stimulus in a positive manner, i.e., performing environmental citizenship behavior. This finding emphasizes the pivotal role of the environmental stimulus in cultivating environmental citizenship behavior among residents in the destinations. In addition, this research also validated the mediating role of relationship quality variables in the impact of perceived ECSR on environmental citizenship behavior. The indirect relationships between perceived ECSR and environmental citizenship behavior suggested that developing a good relationship with the environment can improve residents’ reliance on the destination, which in turn encourages them to adopt environmental citizenship behavior.

### 5.2. Theoretical Contributions

Underpinned by the SOR model, this research investigated how environmental cues (i.e., perceived ECSR) and resident-environment relationship quality influence residents’ environmental citizenship behavior. This contributes to the literature on environmental citizenship behavior in tourism research as follows.

First, this study constructed and examined a theoretical framework based on the SOR model and the relationship quality theory for predicting residents’ environmental citizenship behavior in rural destinations. Since previous research on environmental citizenship behavior has been mainly approached from an employee perspective [11], this study expands the literature on this topic by adding a destination resident perspective in the tourism context. More importantly, this empirical effort indicates a direct pathway from the stimulus (i.e., residents’ perceived ECSR) to the response (residents’ environmental citizenship behavior) and indirect routes through the mediation of resident-environment relationship quality variables (i.e., environmental identification and environmental commitment). The findings of this research further validate the parsimony and robustness of the SOR model through its application in the rural destination context, [33,35]. In summary, this research enriches the literature on the antecedents of environmental citizenship behavior and offers a novel perspective for understanding environmental citizenship behavior in the tourism context [17].

Second, the current research proposed that the relational reciprocity between residents and the destination environment can enhance residents’ perceptions of their identification with destination images and obligation toward the environment, which in turn stimulate environmental citizenship practices. The results of this research established such a connection between residents and destination environment (i.e., enhanced environmental identification and environmental commitment) that facilitated environmental citizenship behavior amongst residents. Relational constructs included in the conceptual framework of the present research were verified to play substantial mediating roles. Despite the evidence in marketing research and tourism research [55,59,60], limited studies have applied environmental identification and environmental commitment to measure relationship quality and predict environmental citizenship behavior [9,27]. By taking both environmental identification and environmental commitment into account, our research demonstrated how residents’ identification with the destination, and commitment to environment conservation, promote residents to adopt citizenship behavior for maintaining environmental sustainability in rural destinations. Therefore, the mediating functions of these relational constructs must be acknowledged by scholars developing or extending the model presented in this research.

Third, this study approached relationship quality from environmental identification and environmental commitment. The correlation between these two sub-dimensions of relationship quality was also investigated. We research showed that as two distinctive notions representing relationship quality, environmental commitment is the positive outcome of environmental identification, which aligns with prior findings [53]. This research thus offers new evidence supporting the positive influence of environmental identification on environmental commitment. By confirming the specific indirect paths from perceived ECSR to environmental citizenship behavior (Table 5), it verifies the mediating role of environmental commitment between the causal relationships among the perceived ECSR, environmental identification and environmental citizenship behavior in the private and public spheres. In sum, it allows us to understand the particular link between two subdimensions of relationship quality, and how they work together to shape environmental citizenship behavior among destination residents.

Lastly, unlike some research treating environmental citizenship behavior as a single-dimensional concept [43], this research described them as a multi-dimensional concept encompassing behavior in the private and public domains. The empirical results indicate that perceived ECSR can also affect residents’ environmental citizenship behavior in private homes in addition to their behavior in the public areas. The results of the mediating effect analysis imply that environmental identification and environmental commitment mediate the relationships between perceived ECSR and two types of environmental citizenship behavior, respectively. This not only supports previous research considering environmental citizenship behavior as a multi-dimensional concept [10], but also confirms that the influence of perceived ECSR on residents‘ environmental citizenship behavior can extend from the public domain to the private domain. Overall, this research expands the body of knowledge on environmental citizenship behavior in different spheres in the tourism context.

### 5.3. Managerial Implications

Empirical findings of this research highlight the importance of perceived ECSR and resident-environment relationship quality in shaping environmental citizenship behavior, which may offer the following practical implications for sustainability of rural destination and destination management.

The results highlight the significance of perceived ECSR as the environmental stimulus in developing environmental citizenship behavior among residents. Destination management organizations should pay close attention to the implementation of ECSR strategies and make it perceivable to residents. For example, Yucun Village broke with its heavy reliance on the former pillar industry–mineral industry, which brought enormous financial advantages but generated severe environmental disruption. Instead, the village launched ecological restoration campaigns and developed rural tourism as the new major industry. Solid waste separation was advocated in the public areas and at home. These measures effectively improved the environmental quality of the village and stimulated residents’ psychological attachment to the place. Consequently, residents who perceived these ECSR practices engaged in sustainable environmental behavior more willingly.

Moreover, our research confirms that relationship quality significantly mediates the relationships between perceived ECSR and environmental citizenship behavior. It suggests that practicing ECSR encourages residents to develop a stronger bond with the destination, which in turn enhances their environmental identification and environmental commitment. As a result, residents are more likely to adopt citizenship behavior for environmental protection. For example, listening to residents’ voices is critical in making tourism development plans, since the implementation of the plans depends on residents’ supports, while these supports come from enhanced environmental identification and environmental commitment among the residents. Interactive community initiatives and environmental awareness events can be planned to showcase how the destination environment and the residents’ well-being are mutually dependent. In addition, strategies can be made to establish a smooth communication channel between the destination management and residents. These efforts improve resident-environment relationship quality which further facilitates the adoption of environmental citizenship behavior amongst residents.

Lastly, the division of the private-sphere and public-sphere environmental citizenship behavior implies that environmental citizenship behavior in these two domains are equally important. Destination managers have to understand that the evaluation of residents’ environmental citizenship behavior should be approached not merely from the public domain, but also the private one. Thus, they can gain a more comprehensive understanding of how to measure residents’ environmental citizenship behavior, providing new insights into making strategies to encourage residents’ sustainable behavior. In addition, destination managers should take measures to leverage perceived ECSR and relationship quality variables to influence residents’ environmental citizenship behavior both in the public and private spheres.

## 6. Limitations and Future Research

This research had several limitations which may offer opportunities for future research. First, relationship quality was divided into environmental identification and environmental commitment. Future research may classify them into other subdimensions for a more detailed and holistic understanding of how relationship quality variables affect environmental citizenship behavior. Second, the data were collected from one site to validate the conceptual model, which may restrain its stability and generalizability. There were two main reasons for doing so. One the one hand, considerable studies on destination residents have used data obtained from a single study site and produced valuable empirical findings, which validates the efficacy of this approach [88,89,90,91,92]. On the other hand, since living at a place permanently usually results in residents having more concerns when answering the questions, a door-to-door field survey is more difficult for the researchers compared to the survey of tourists. Particularly, the potential risks posed by the coronavirus and epidemic prevention and control measures add more difficulties at present. Thus, cross-validation with other sites in different types of destinations (e.g., urban destinations) is recommended when the conditions for the field survey improve in the future. Third, as cross-sectional data were collected for this research, they may not comprehensively capture the causal relationships in this theoretical framework. Longitudinal data are thus suggested to re-examine whether the effects presented in this research are durable in multiple timeframes [27]. Lastly, the SEM method was adopted to examine the proposed hypotheses. However, this method is only applicable to symmetrical causal relationships. Recent research has involved the application of fuzzy-set qualitative comparative analysis (fsQCA) for asymmetrical relationships among variables [55]. Other interesting approaches should also be considered for future research, such as experimental methods [93,94] and a hybrid method, such as self-administrated questionnaires and semi-structural interviews [95,96].

## Figures and Tables

**Table 1:** Measurement items.

Construct	Item	Source
Perceived Environmental CSR (ECSR)	ECSR1	[11]
	ECSR2	
	ECSR3	
	ECSR4	
Environmental identification (EI)	EI1	[27]
	EI2	
	EI3	
Environmental commitment (EC)	EC1	[27]
	EC2	
	EC3	
	EC4	
Private-sphere ECB	PRECB1	[10]
	PRECB2	
	PRECB3	
Public-sphere ECB	PUECB1	[10]
	PUECB2	
	PUECB3	

Note: See Table A1 in Appendix A for detailed measurements of all variables.

**Table 2:** Assessment of measurement model.

Construct and Item	Std. Factor Loading	*t* Values	Composite Reliability	Average Variance Extracted	Alpha
ECSR			0.911	0.719	0.909
ECSR1	0.829	16.793			
ECSR2	0.866	17.862			
ECSR3	0.872	18.05			
ECSR4	0.823	-			
EI			0.801	0.576	0.784
EI1	0.615	10.061			
EI2	0.845	12.103			
EI3	0.798	-			
EC			0.902	0.698	0.902
EC1	0.739	15.125			
EC2	0.819	17.797			
EC3	0.907	20.869			
EC4	0.867	-			
PRECB			0.860	0.673	0.842
PRECB1	0.837	13.495			
PRECB2	0.893	13.839			
PRECB3	0.721	-			
PUECB			0.786	0.553	0.776
PUECB1	0.825	11.896			
PUECB2	0.637	10.037			
PUECB3	0.757	-			

**Table 3:** Discriminant validity assessment.

Construct	ECSR	EI	EC	PRECB	PUECB
ECSR	0.848				
EI	0.373	0.759			
EC	0.422	0.348	0.835		
PRECB	0.451	0.355	0.474	0.820	
PUECB	0.591	0.409	0.403	0.385	0.744

**Table 4:** Structural model analysis and hypothesis test result.

Hypotheses	Path	Standardized Coefficient	*t* Values	Results
H1	ECSR→EI	0.373	5.582 ***	Supported
H2	ECSR→EC	0.339	5.215 ***	Supported
H3	EI→EC	0.222	3.311 ***	Supported
H4	EI→PRECB	0.149	2.241 *	Supported
H5	EI→PUECB	0.188	2.76 **	Supported
H6	EC→PRECB	0.311	4.641 ***	Supported
H7	EC→PUECB	0.144	2.203 *	Supported
H8	ECSR→PRECB	0.266	3.959 ***	Supported
H9	ECSR→PUECB	0.462	6.384 ***	Supported

Note: * *p* < 0.05, ** *p* < 0.01, *** *p* < 0.001.

**Table 5:** Mediation test results.

Mediating Hypothesized Path	Indirect Effects	95% Bias-Corrected Confidence Intervals			95% Percentile Confidence Intervals			Results
		Lower	Upper	*p*-Value	Lower	Upper	*p*-Value	
ECSR→EI→PRECB	0.056	0.009	0.123	0.022	0.005	0.117	0.030	Supported
ECSR→EI→EC→PRECB	0.026	0.008	0.063	0.002	0.006	0.057	0.005	Supported
ECSR→EC→PRECB	0.107	0.056	0.179	0.000	0.051	0.171	0.000	Supported
ECSR→EI→PUECB	0.072	0.020	0.137	0.008	0.018	0.135	0.010	Supported
ECSR→EI→EC→PUECB	0.012	0.002	0.035	0.014	0.001	0.030	0.032	Supported
ECSR→EC→PUECB	0.050	0.008	0.112	0.016	0.004	0.104	0.027	Supported

## Data Availability

Not applicable.

## Appendix A

**Table A1:** Detailed measurements of all variables.

Construct	Item	Source
Perceived Environmental CSR (ECSR)	ECSR1 This destination implements special programs to minimize its negative impact on the natural environment.	[11]
	ECSR2 This destination participates in activities which aim to protect and improve the quality of the natural environment.	
	ECSR3 This destination targets sustainable growth which considers future generations.	
	ECSR4 This destination makes investments to create a better life for future generations.	
Environmental identification (EI)	EI1 I am very interested in what others think about the natural environment of this destination.	[27]
	EI2 When someone praises the environment of this destination, it feels like a personal compliment	
	EI3 When someone criticizes the environment of this destination I would feel embarrassed	
Environmental commitment (EC)	EC1 In my mind, I am committed to maintaining the greatest environmental interests of this destination.	[27]
	EC2 I strongly feel that I am closely related to the environment of this destination.	
	EC3 In the future, I will be interested in strengthening the connection with the environment of this destination.	
	EC4 I am expecting a strong connection with the environment of this destination.	
Private-sphere ECB	PRECB1 At home, I reduce the amount of energy I use.	[10]
	PRECB2 I recycle cans, bottles and papers.	
	PRECB3 I buy products that are friendly to the environment.	
Public-sphere ECB	PUECB1 I vote for a candidate or referendum that supports environmental protection.	[10]
	PUECB2 I keep the surrounding environment clean.	
	PUECB3 I encourage people around me joining and donating to environmental organizations.	
